# A bi-specific CAR-T cell therapy targeting CD19 and CD22 in relapsed or refractory B-ALL

**DOI:** 10.1007/s10238-025-01637-8

**Published:** 2025-07-28

**Authors:** Qiuling Ma, Runhong Wei, Qingming Wang, Songfu Jiang, Yi Wu, Chao Min, Shufang Guo, Yu Zhang, Xiaohong Sun, Haigang Wu, Xuedong Sun, Fang Xiang, Mingxing Xiao, Zhi Cheng

**Affiliations:** 1 The Second School of Clinical Medicine, Henan University of Chinese Medicine, Zhengzhou, 450046 Henan Province China; 2 Department of Hematology, Henan Province Hospital of Traditional Chinese Medicine (the Second Affiliated Hospital, Henan University of Chinese Medicine), Institute of Hematology, Henan University of Chinese Medicine, Zhengzhou, 450002 Henan Province China; 3 The 2nd Affiliated Hospital of Nanchang University, Jiangxi Provincial Key Laboratory of Hematological Diseases, Nanchang, 330006 Jiangxi Province China; 4 Department of Hematology, The First Affiliated Hospital of Wenzhou Medical University, Wenzhou, 325000 Zhejiang Province China; 5 He Nan Province Engineering Research Center of Integrative Medicine for the Prevention and Treatment of Hematological Diseases, Zhengzhou, China; 6 School of Life Sciences, Henan University, Kaifeng City, 475000 China; 7 Hrain Biotechnology Co., Ltd., Shanghai, 200030 China

**Keywords:** Chimeric antigen receptor T cells, R/r B-ALL, Bi-specific CAR, CD19, CD22

## Abstract

CAR-T therapies targeting either CD19 or CD22 have shown significant promise for treating relapsed or refractory B-lineage acute lymphoblastic leukemia (r/r B-ALL). However, a common limitation is the high frequency of antigen loss, which leads to r/r B-ALL progression. To overcome progression caused by antigen loss, bi-specific CAR-T immunotherapies targeting both CD19 and CD22 may offer enhanced efficacy in eliminating r/r B-ALL and preventing relapse by hindering leukemia cell proliferation. In this study, we present both pre-clinical and clinical findings from an ongoing trial (NCT04303520) assessing the therapeutic efficacy of the CD19-CD22 bi-specific CAR-T therapy for r/r B-ALL patients. Pre-clinical data from animal models reveal that CD19-CD22 CAR-T cells effectively induce cytotoxicity, thus suppressing B-ALL cell proliferation. Notably, this dual-targeted approach outperforms single-target CAR-T treatments. From the Phase I trial encompassing 35 participants, 37.1% (13 out of 35 patients) experienced cytokine release syndrome, with only a single case of Grade III   severity. Importantly, no neurotoxicity episodes were recorded post CD19-CD22 CAR-T administration. Common adverse events included hematological, gastrointestinal, and nutrition-related disturbances. B-ALL patients undergoing stem cell transplantation in tandem with CAR-T therapy exhibited a pronounced improvement in overall survival compared to those treated solely with CAR-T. The median overall survival duration was 21.49 ± 4.4 months (95% CI: 14.31–31.40 months), meanwhile one-year PFS and OS are 0.37 (95% CI, 0.21–0.49) and 0.62 (95% CI: 0.52–0.71), respectively. Clinical monitoring did not identify significant side effects associated with the CD19-CD22 regimen. To monitor the inflammation-associated factors accompanying CAR-T therapy, the peak value of CAR DNA copies was reached around Day 10, accompanied by a similar trend in the levels of inflammatory factors, including IFN-*γ*, Granzyme B, IL-6, and CRP. Collectively, our data advocate for the potential role of bi-specific CD19-CD22 CAR-T therapy in addressing r/r B-ALL.

*Trial registration number* ClinicalTrials.gov (No. NCT04303520).

**Supplementary Information:**

The online version contains supplementary material available at 10.1007/s10238-025-01637-8.

## Introduction

In patients with r/r B cell acute lymphoblastic leukemia (B-ALL), outcomes following chemotherapy are often suboptimal [1]. After two cycles of intensive multiagent consolidation therapy, the 5-year relative survival for B-ALL patients ranges between 25 and 50% [2, 3]. To enhance clinical outcomes, emerging therapeutic strategies such as blinatumomab [4, 5], natural killer cells [6, 7], and chimeric antigen receptor T cell (CAR-T) therapy [8, 9] have been introduced. Compared with other therapeutics, the CAR-T therapy displayed the remarkable success in the malignancy treatment.

Bi-specific chimeric antigen receptor T (CAR‐T) cells offer a promising strategy to overcome drug resistance and improve clinical outcomes relative to single‐target CAR‐T therapies. Therapeutic failure in CAR‐T applications is multifactorial, arising from antigen loss or downregulation, limited CAR‐T cell persistence, treatment‐related toxicities, and tumor heterogeneity. In response, dual‐targeted CAR‐T cells have been developed to address tumor heterogeneity by recognizing distinct antigens, thereby enabling the eradication of diverse tumor subpopulations and reducing the likelihood of therapeutic escape. For example, Heng Mei et al. reported that BCMA-specific CAR-T targeting BCMA and CD38 presented the significant therapy efficacy in refractory or relapsed multiple myeloma [10]; Eugenia Zah et al. reported that bi-specific CAR-T targeting CD319 and B cell maturation antigen (BCMA) can robustly suppress proliferation of heterogeneous multiple myeloma [11]; Yuanyan Tang et al. reported that bi-specific CAR-T targeting CD38 and BCMA displayed the advanced therapy outcomes compared to single-targeting CAR-T in vivo [12]; Dian Zhou et al. reported that anti-BCMA/GPRC5D bi-specific CAR-T cells showed higher safety in human body and encouraging activity in relapsed or refractory multiple myeloma [13].

Among these emerging therapeutic targets, the current CAR-T therapies targeting CD19 have shown promising therapeutic efficacy in leukemia, particularly for r/r B-ALL [14–16]. However, a recurrent challenge is the loss of CD19 expression in B-ALL, leading to diminished effectiveness of anti-CD19 CAR-T therapies, despite their standardization in leukemia treatment [17–19]. Intriguingly, CD22 is often expressed in most r/r B-ALL patients, especially after CD19 loss due to CAR-T therapy [20, 21]. Numerous pre-clinical and clinical studies have affirmed the anti-tumor efficacy of targeting CD22 in leukemia [22, 23]. Clinical interventions with CD22 CAR-T have effectively hindered leukemia cell proliferation in vivo [15, 24]. Similar to CD19-targeted therapies, CD22 CAR-T treatments face resistance due to down-regulation or loss of CD22, allowing B-ALL to circumvent the treatment [25–28]. Existing literature suggested that simultaneous down-regulation of both CD19 and CD22 in leukemic cells is rare. This presents a compelling case for dual-target therapies as a potential means to overcome CAR-T resistance [14, 15, 25]. Indeed, dual-targeting CAR-T therapies focusing on both CD19 and CD22 have demonstrated substantial clinical benefits in B-ALL [26, 27, 29]. Several methodologies exist for dual CAR targeting, including CAR-T cell mixtures [30], co-transducing T cells with two separate vectors [16], and utilizing bicistronic vectors for T cell transfection [24, 31]. By engineering a bicistronic CAR vector integrated with both anti-CD19 and anti-CD22 components, there is a potential for r/r B-ALL patients to achieve long-term complete remission.

In this multicenter study, we engineered a bi-specific CAR construct targeting both CD19 and CD22. We assessed the CAR-T functionality through infection efficiencies, cytokine release profiles, and effector-to-target ratios against a spectrum of B-ALL cell lines, each with varying CD19 or CD22 expression. The in vivo antitumor efficacy of the bi-specific CD19-CD22 CAR-T cells was evaluated using a Nalm6-bearing B-ALL xenograft model, benchmarked against negative controls and single-target CD19 or CD22 groups. Drawing from the insights of our preclinical findings, we initiated a Phase I clinical trial employing the CD19-CD22 CAR-T for r/r B-ALL treatment, enrolling 35 patients. We subsequently analyzed the trial data to gauge clinical benefits, monitor adverse events, and delineate baseline patient characteristics associated with the CD19-CD22 CAR-T therapy.

## Results

### CD19-CD22 CAR-T cells exhibit antitumor activity in pr-eclinical B-ALL xenograft model

Earlier studies have demonstrated that single-target CAR-T therapies, such as those targeting the CD19 antigen, often confront resistance due to CD19 antigen loss, while CD22 remains stably expressed in relapsed B-ALL patients (SI Fig. S1) [15]. To address this therapeutic challenge, we devised a bi-specific CAR-T targeting both CD19 and CD22 for r/r B-ALL patients.

**Fig. 1:**
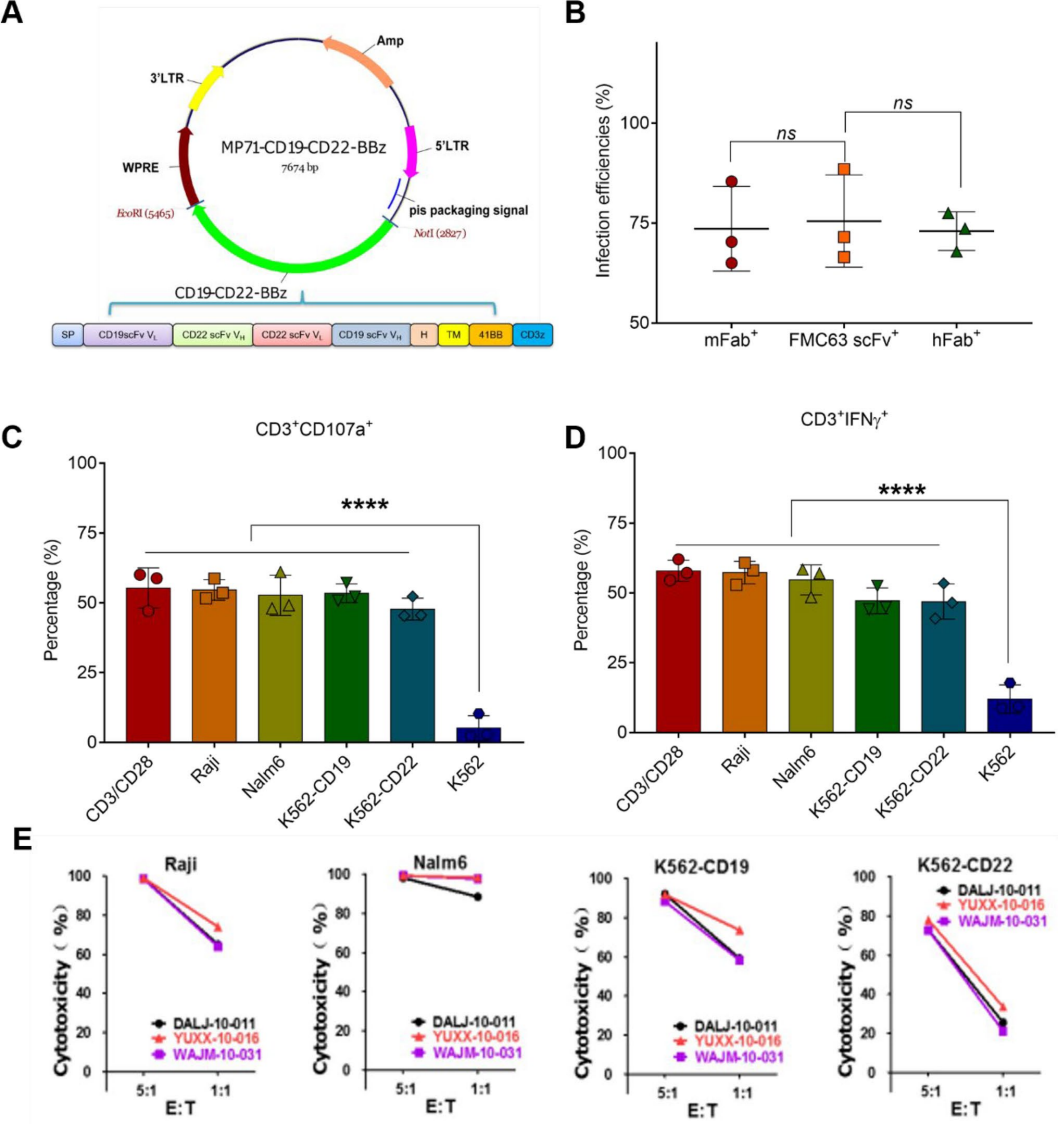
**In vitro cytotoxicity of bi-specific CD19-CD22 CAR-T cells. A** Schematic representation of the bi-specific anti-CD19/CD22 CAR constructs with CD19/CD22 or BBz co-stimulatory signaling domains. **B** CAR fragment infection efficiencies assessed via flow cytometry using cells from three distinct donors. **C**, **D** Detection of CD107a and IFN-*γ* production in CD3 + T cells (utilizing CD3 + /CD28 + T cells, Raji, Nalm6, K562-CD19, K562-CD22, and K562 cell lines) following treatment with CD19-CD22 CAR-T cells at a 1:1 ratio. **E** Evaluation of in vitro cytotoxicity exhibited by CD19-CD22 CAR-T cells against Raji, Nalm6, K562-CD19, and K562-CD22 cell lines at varying effector-to-target (E:T) ratios of 5:1 and 1:1. Data were represented as Means ± SEM. *P*-value was calculated using one-way ANOVA with multiple comparison: ns, no significance; ****, *p* < 0.0001

In this study, our first step was to engineer a bicistronic CAR-T vector encoding the dual-target CAR molecules for CD19 and CD22 (Fig. 1A). To ascertain the functionality of this vector, primary T cells were sourced from three distinct human donors, showing minimal impact on targeted cell proliferation (SI Table S1). Subsequent transduction assays were carried out to evaluate the efficiency of CAR vector transduction and fusion. After infection, we assessed the relative protein expression in primary blood mononuclear cells (PBMCs) via flow cytometry, as detailed in the Materials and Methods section. As depicted in Fig. 1B, approximately 75% of T cells from patient donors exhibited expression of CAR constructs with signaling domains, including mFab + , FMC63 scFV + , and hFab + fragments. Notably, in vivo expansion of these fragments did not display significant differences among them. These findings suggest the successful generation of therapeutic CAR-T cells using our bicistronic construct.

Cytokines, notably interferon (IFN)-γ and CD107a, are critical for T cell activation and exert antileukemic effects in vivo. Their levels serve as key indicators of CAR-T cell functionality. We therefore assessed the secretion of IFN-γ following incubation with various cancer cells including Raji, Nalm6, K562-CD19, K562-CD22, CD3 + /CD28 + T cells, and K562. As depicted in Fig. 1C, D, the proportions of CD3 + /CD107a + and CD3 + /IFN-γ + cells within the cell groups Raji, Nalm6, K562-CD19, and K562-CD22 were approximately 55%, mirroring the levels observed with the positive control, CD3 + /CD28 + T cells, with no significant difference (SI Figs. S2, S3). Additionally, these expression efficiencies in targeted cell lines markedly surpassed those of the negative control, the K562 cell line. Subsequently, we evaluated the cytotoxic activity and effector-to-target (E:T) ratios of CD19-CD22 CAR-T cells against the targeted cell lines. As illustrated in Fig. 1E, CAR-T cells from three individual donors demonstrated significant cytotoxicity against the target cell lines in a ratio-dependent manner.

**Fig. 2:**
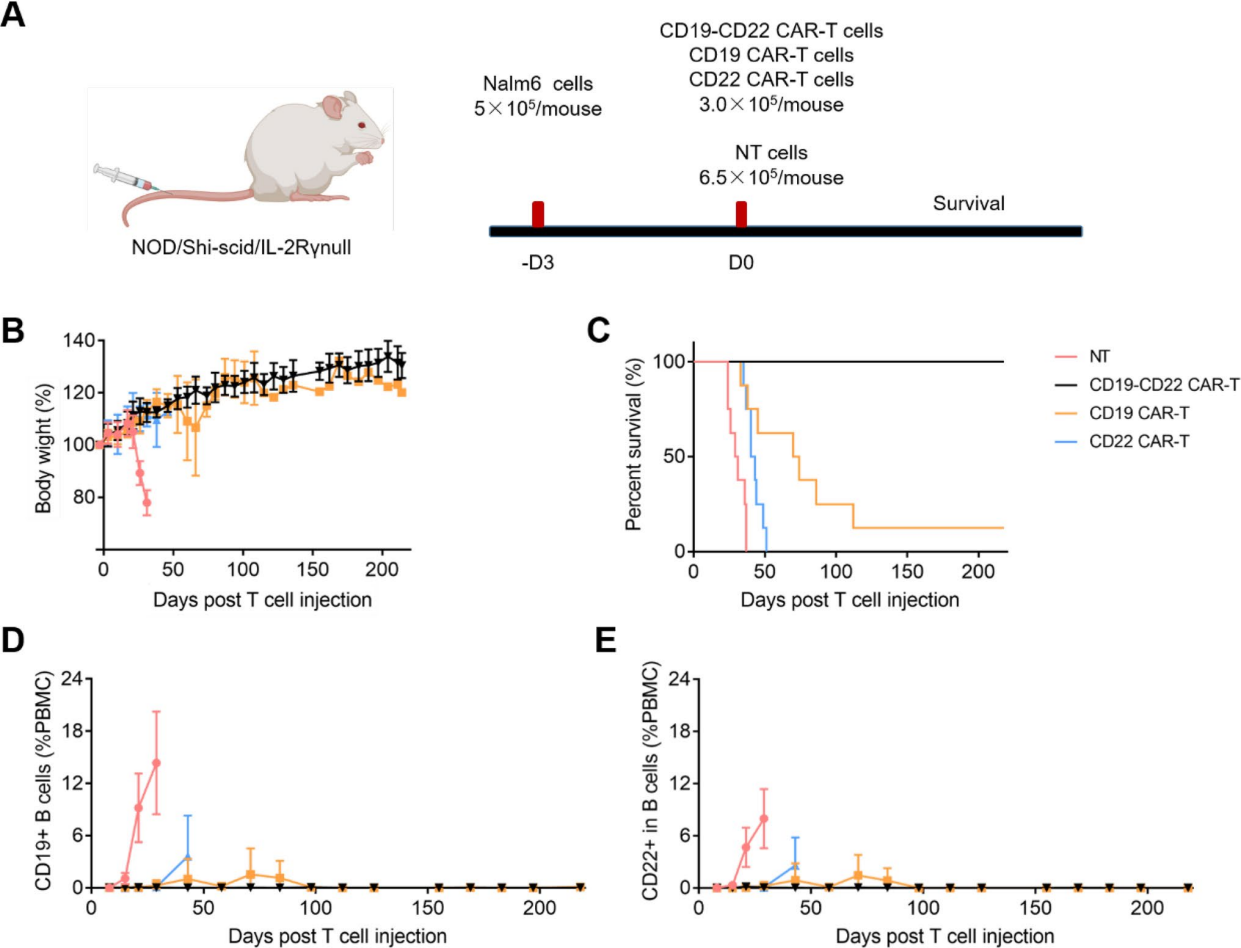
**Eradication of Nalm6 by bi-specific CD19-CD22 CAR-T cells in a B-ALL xenograft model.** **A** Diagrammatic representation of the Nalm6 xenograft model in NOG mice and the associated therapeutic strategy. **B** Temporal progression of body weight in NOG mice post-treatment: Negative control (NT, pink curve), CD19 CAR-T cells (yellow curve), CD22 CAR-T cells (blue curve), and CD19-CD22 CAR-T cells (black curve). **C** Kaplan–Meier survival analysis for mice treated with CD19 CAR-T cells, CD22 CAR-T cells, and CD19-CD22 CAR-T cells (*n* = 8 mice per group). The median survival post-treatment was observed to be 30 days (NT group), 41.5 days (CD22 CAR-T therapy), 72 days (CD19 CAR-T therapy), and not determined (n.t.) for the CD19-CD22 CAR-T therapy group. **D** Longitudinal analysis of circulating CD19 + B cells using flow cytometry following treatments with CD19 CAR-T cells, CD22 CAR-T cells, and CD19-CD22 CAR-T cells. **E** Temporal profile of circulating CD22 + B cells determined by flow cytometry post-treatments with CD19 CAR-T cells, CD22 CAR-T cells, and CD19-CD22 CAR-T cells. The color coding in Fig. 2D, E corresponds to that in Fig. 2B, C. Two-way ANOVA with Sidak’s multiple comparisons test. n.d. not detection. Data are represented as Means ± SEM

To assess the antitumor efficacy of CD19-CD22 CAR-T cells in vivo, we utilized Nalm6 cells to establish a B-ALL xenograft model through tail vein injection. Subsequently, these mice received treatment with bi-specific CD19-CD22 CAR-T cells, with comparisons drawn to respective control groups, namely the negative control, CD19 CAR-T cells, and CD22 CAR-T cells (Fig. 2A). Following intravenous (*i.v.*) administration of CAR-T cells, the body weight of all groups typically increased until Day 6. However, a pronounced decline in body weight was noted in the negative control group, attributed to the in vivo proliferation of Nalm6 cancer cells (Fig. 2B). An analysis of median survival revealed that the CD19-CD22 CAR-T group exhibited complete remission extending up to Day 218. This starkly surpassed the median survival observed in the CD19 CAR-T (72 days, *p* = 0.0004), CD22 CAR-T (41.5 days, *p* < 0.0001), and the negative control groups (30 days, *p* < 0.0001) (Fig. 2C). To delve deeper into leukemia cell proliferation post-injection, we noted that the percentage of CD19 or CD22 positive peripheral blood (PB) cells following CD19-CD22 CAR-T treatment was markedly lower than in other groups (Fig. 2D, E). Moreover, the population of CD19 or CD22 positive PB cells in the CD19 or CD22 CAR-T treatment groups surged between Day 58 and Day 71, potentially contributing to mouse mortality. Taken together, these results suggest that bi-specific CD19-CD22 CAR-T cells effectively curtail cancer cell proliferation in the Nalm6 xenograft model.

### Characterization of enrolled patients in CD19-CD22 CAR-T therapy

Between February 2018 and February 2021, 35 patients with refractory/relapsed B-ALL were enrolled and treated with bi-specific CD19-CD22 CAR-T cells. Patient characteristics are summarized in SI Table S1. Ages ranged from 4 to 60 years, with a median of 26 years. Based on an age threshold of 21 years, patients were categorized into a younger group (*n* = 16, median age 14 years) and an older group (*n* = 19, median age 43 years). Critical characteristics, including ECOG score, gene mutation, stem cell transplants, juvenile cell abundance, and CD19 or CD22 expression levels, are detailed in SI Table S2. Notably, only one patient had undergone allogeneic hematopoietic stem cell transplantation prior to CAR-T therapy. Over half of the patients had ECOG scores of 0–1 (*n* = 22, 62.86%), with a balanced distribution of patients in the low ECOG score range (0–1). Seven B-ALL samples were positive for the Philadelphia chromosome, and 12 had gene mutations. Twenty-two original B-ALL samples exhibited a juvenile cell abundance exceeding 20%. Prior to CAR-T cell infusion, 8 patients were CD19 + /CD22-, one was CD19-/CD22 + , and 20 were CD19 + /CD22 + .

### Adverse events post CAR-T therapy

**Fig. 3:**
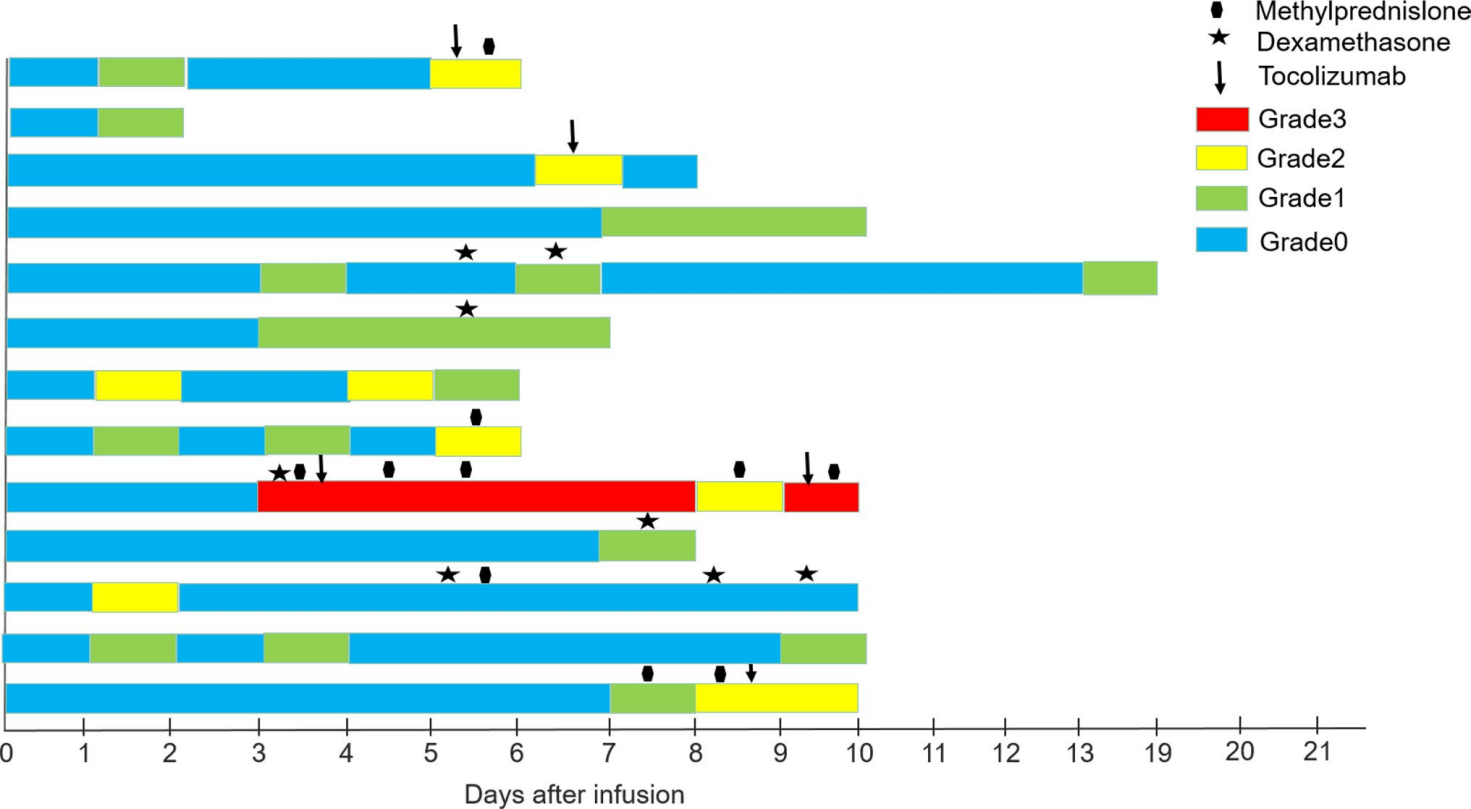
**Swimmer plot illustrating CRS onset and duration in 13 patients post CD19-CD22 CAR-T cell infusion. **Median CRS onset: Day 3 (range: Day 1–7); median CRS duration: 4 days (range: 1–11 days)

Adverse events occurring within 30 days post CAR-T treatment were meticulously recorded and graded based on a prior study [32]. Comprehensive assessments covered various systems and included parameters such as cytokine release syndrome (CRS), blood indices, infections, and evaluations of the respiratory, digestive, ocular, immune, vascular, neurological, cardiac, hepatic, skeletal, renal systems, as well as nutrient and metabolism-related disorders. Detailed event profiles are available in SI Tables S3–S5. Focusing on CRS, 41.94% (13/31, 4 patients were not detected) of patients exhibited CRS symptoms post-infusion (SI Table S3). Notably, only one patient reached Grade-3 severity. The CRS response timeline and intervention strategies for these patients are illustrated in Fig. 3. The median onset and duration of CRS were Day 3 and 4 days, respectively. To mitigate CRS, interventions like methylprednisolone, dexamethasone, and tocolizumab were administered. Despite these measures, CRS symptoms persisted in all but two patients, suggesting that CRS could be a primary adverse outcome of CAR-T cell therapy. The lone patient with Grade-3 CRS showed progressive disease, and subsequent tracking was lost. Interestingly, cytokine release encephalopathy syndrome (CRES) was absent among the treated r/r B-ALL patients, underscoring the reduced toxicity of the bi-specific CD19-CD22 CAR-T cells. Concerning other adverse events, blood circulation-related reactions were predominant, aligning with previous studies [33, 34].

### Anticancer responses

**Fig. 4:**
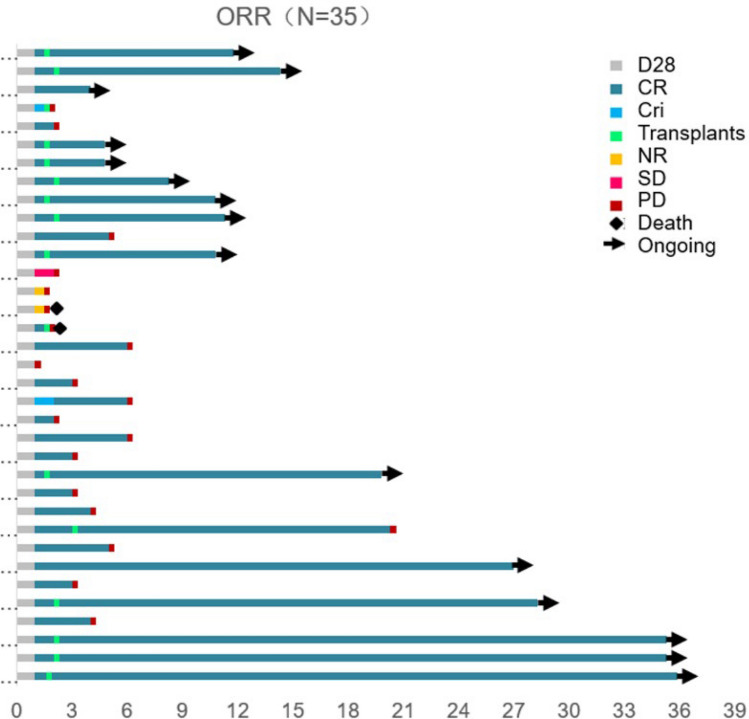
**Swimmer plot for the 35 patients post CD19-CD22 CAR-T cell infusion.** Key: *CR* complete remission; *CRi* complete response with incomplete hematologic recovery; *NR* no response; *SD* stable disease; *PD* progressive disease

**Fig. 5:**
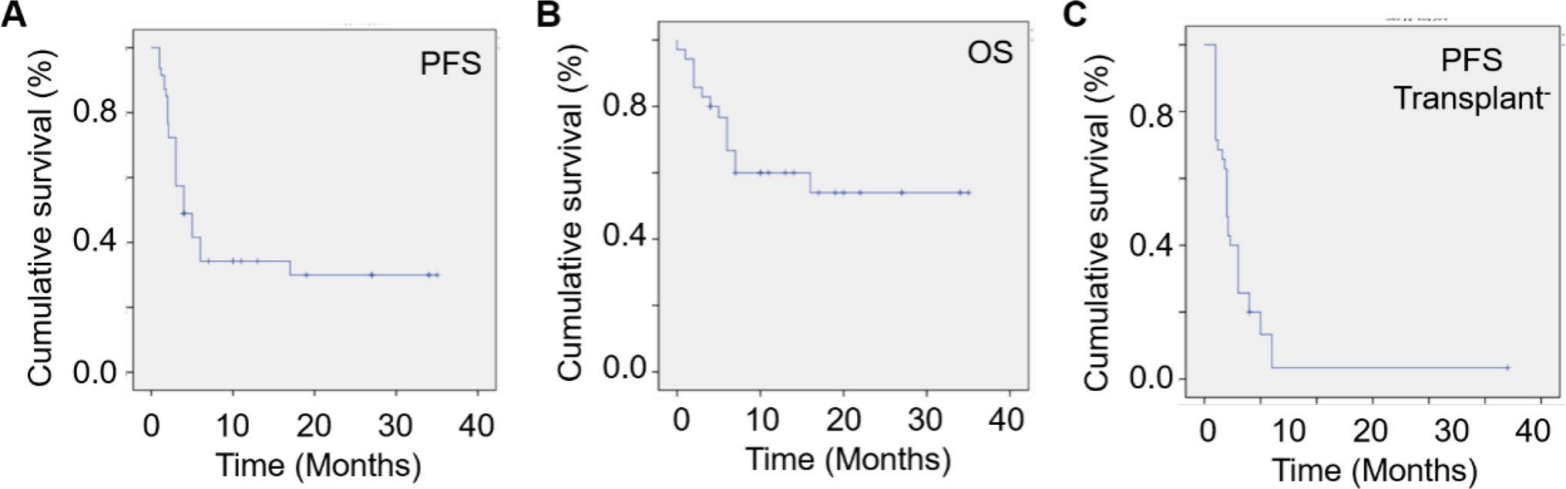
**Clinical outcomes post bi-specific CD22-CD19 CAR-T cell infusion.** **A** Progression-free survival post CD22-CD19 CAR-T therapy for B-ALL patients. Median survival is 4.00 ± 0.92 months, 95% CI (2.20–5.80). **B** Overall survival post CD22-CD19 CAR-T therapy for B-ALL patients. Average survival is 21.49 ± 4.4 months, 95% CI (14.31–31.40). **C** Progression-free survival post CD22-CD19 CAR-T therapy for B-ALL patients without stem cell transplantation. Median survival is 2.0 ± 0.13 months, 95% CI (1.75–2.25). Data were estimated using Kaplan–Meier method for 31-patient cohort. Overall response rate (ORR) is equal to 88.57% (31/35), 95% CI (78.8–98.4%) with 42.86% CRS rate (15/35). ORR in 3 month is 50% (10/20), 95% CI (28.4–71.6%) with 50% CRS rate (10/20). Better ORR in 3 month is 80% (16/20), and 95% CI (62.4–97.6%) with 50% CRS rate (10/20)

In the CAR-T cell therapy study, the dosage administered to B-ALL patients were determined by their therapeutic group (not presented). The intervention dosages were as follows: 21 patients received the intervention dosage is 6 × 10^6^ cell/kg, intervention dosage of 2 patients is 1.2 × 10^7^ cell/kg, intervention dosage of 2 patients is 8 × 10^6^ cell/kg, intervention dosage of 1 patient is 4 × 10^6^ cell/kg, intervention dosage of 3 patients is 3 × 10^6^ cell/kg, intervention dosage of 4 patients is 2 × 10^6^ cell/kg, and intervention dosage of 3 patients is 1 × 10^6^ cell/kg. Post-treatment outcomes were illustrated in Fig. 4. Four patients were excluded from the study: two who did not achieve no response (NR), one who passed away, and one who maintained stable disease (SD). One month after CAR-T infusion, 82.86% (29/35) of patients achieved complete remission (CR) as shown in Fig. 4. Of these, 45.71% (16/35) underwent stem cell transplantation. Of those who didn’t undergo transplantation, 2/15 patients were maintained CR to the present day. Among those who received the transplantation, 81.25% (13/16) still remain in CR. The average overall survival stood at 21.49 ± 4.4 months (95% CI: 14.31–31.40). The median progression-free survival (PFS) for all patients was 4.00 ± 0.92 months (95% CI: 2.20–5.80), but it decreased to 2.0 ± 0.13 months (95% CI: 1.75–2.25) for those without stem cell transplantation. Stem cell transplantation enhances CAR‐T cell therapy by promoting immune reconstitution and modulating the tumor microenvironment, thereby facilitating CAR‐T cell expansion, persistence, and antitumor activity. Additionally, it restores hematopoietic function following high-dose chemotherapy or radiotherapy, mitigating treatment-related toxicity and contributing to improved therapeutic outcomes. A separate analysis was also conducted based on patient age (with a threshold of 21 years). To analyze the one-year PFS and OS (Fig. 5), we observed that is about 0.37 (95% CI, 0.21–0.49) and 0.62 (95% CI: 0.52–0.71), respectively. This subgroup analysis did not show any significant deviation from the outcomes observed in the total patient cohort, as evidenced in SI Fig. S4 and Table S7.

**Fig. 6:**
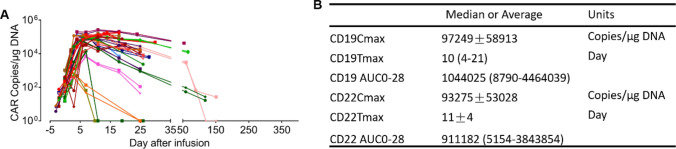
In vivo **expansion kinetics of CD19 or CD22 CAR copies in patients after autologous T cell infusion.** **A** Time-course peak CAR copies of CD19 or CD22 CAR assessed based on the total DNA in the peripheral blood (PB). **B** Expansion kinetic analysis of CAR molecules in patients’ blood. Data were represented as Mean ± Standard Error

Using qPCR, we assessed the in vivo expansion kinetics of either CD19 or CD22 CAR copies in the peripheral blood (PB) of 31 patients with post-treatment (excluding patient without continuous tracking). Fig. 6A shows that most B-ALL patients who underwent CAR-T cell infusion exhibited detectable levels of circulating CAR-T cells. The peak abundance of CD19 or CD22 CAR copies was noted around Day 10, as depicted in Fig. 6B. Further, analyzing the area under the curve (AUC) for the CD19 or CD22 CAR copies in PB revealed comparable expansion kinetics for both CD19 and CD22 over a 28-day period.

**Fig. 7:**
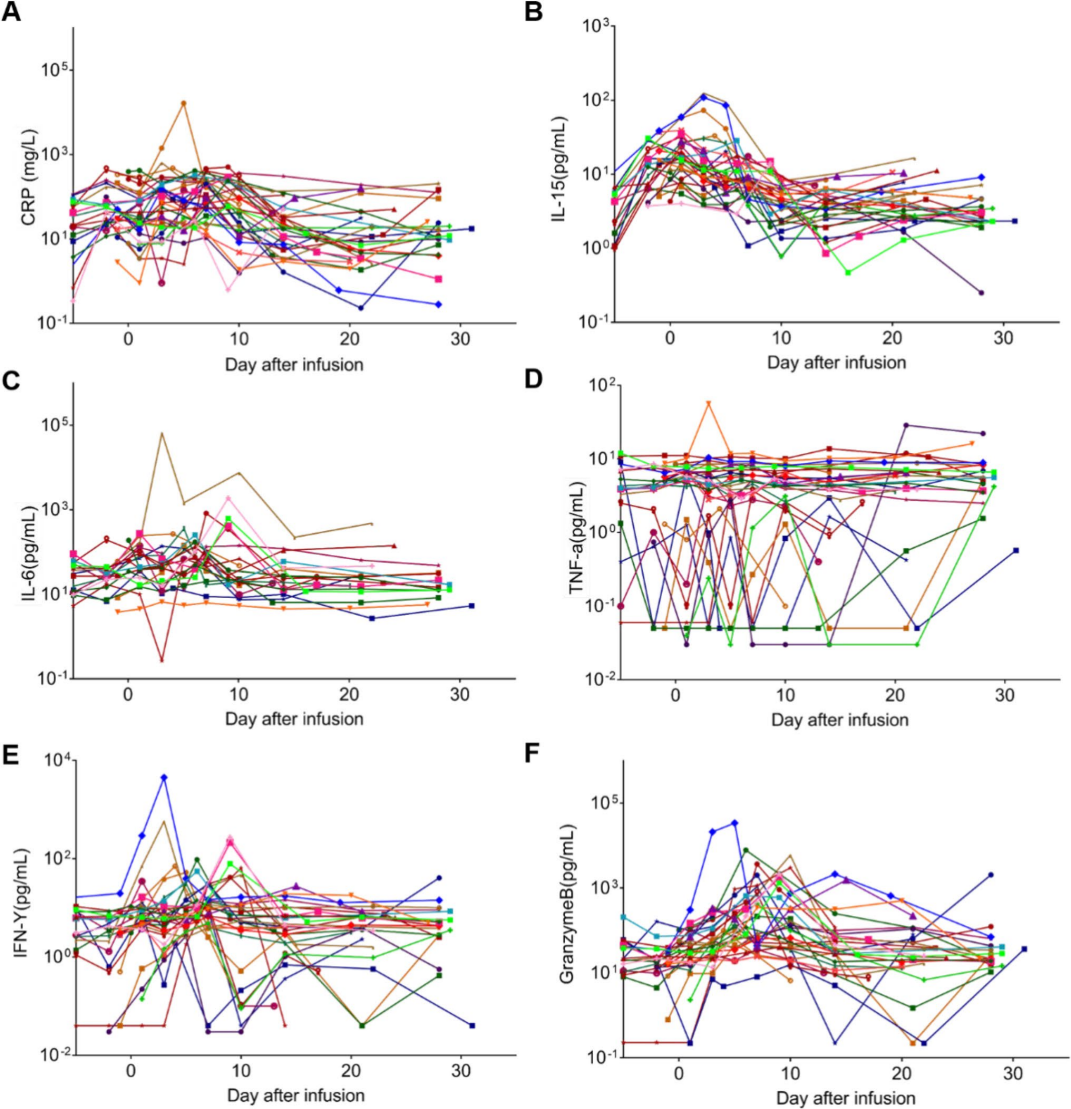
**Cytokine production by r/r B-ALL patients post receiving CD19/CD22 CAR-T therapy. A** C-reactive protein (CRP): **B** IL-15; **C** IL-6; **D** TNF-*α*; **E** IFN-*γ*; **F** Granzyme B

In assessing potential side effects from CAR-T therapy, we studied several factors associated with CRS, such as CRP, IL-15, IL-6, TNF-*α*, IFN-*γ*, and Granzyme B. According to Fig. 7, most of these factors showed a peak around Day 10 post-infusion, apart from IL-15 and TNF-*α*. Notably, TNF-*α* didn’t demonstrate a time-dependent response in many patients with post-infusion. Meanwhile, IL-15 peaked around Day 5, which aligned with the expansion of CD19 or CD22 CAR copies. This is in line with a previous study that linked IL-15 with lymphoma remission following CAR-T therapy [29].

To ascertain which factors might influence the effectiveness of the CAR-T therapy, we looked into baseline characteristics of patients and how these correlated with complete remission (CR). From our data (SI Table S2), certain baseline traits like gender, age, presence of CD19 or CD22, bone marrow blasts, Philadelphia chromosome presence, and ECOG score didn’t significantly affect the chances of achieving CR. However, after delving deeper into post-infusion characteristics (SI Table S6), we discovered that CD19_AUC0-28_, CD22_Cmax_, CD22_AUC0-28_, and IFN-*γ*_Tmax_ had a positive correlation with CR. This suggests these factors might serve as predictive markers for CAR-T therapy’s effectiveness in r/r B-ALL patients.

## Discussion

Our study examined the impact of the dual-targeted CD19-CD22 CAR-T cell therapy on treating relapsed/refractory B-cell acute lymphoblastic leukemia (r/r B-ALL). We found that this therapy effectively combats leukemia, offers prolonged benefits, and is safer with fewer side effects.

While anti-CD19 CAR-T therapy has significantly prolonged the median survival of r/r B-ALL patients [35], many of these patients subsequently face disease progression, predominantly due to therapeutic resistance stemming from antigen loss [14]. Addressing this resistance, dual-specific target therapies have emerged to suppress B-ALL proliferation in vivo. In recent years, early-phase trials of anti-CD19 CAR-T cells have shown promising remission rates in r/r B-ALL patients. However, relapses often occur due to CD19 antigen loss in progressed patients [15, 36, 37]. To overcome this single-target loss resistance, strategies involving multiple co-targeting CAR-T cells have been explored, potentially reducing the chance of gene-loss relapse. We thus present a bi-specific CD19-CD22 CAR-T approach using a bicistronic CAR vector, with its antiproliferative effects assessed in the Nalm6 xenograft model (Fig. 1A). To ascertain the antitumor potential of the CD19-CD22 bi-specific CAR-T for r/r B-ALL, we constructed the corresponding CAR plasmid (Fig. 1A). Notably, transfection and expression levels of key indices, such as mFab + FMC63 scFv + , and hFab + in T cells, were not influenced by specific cell line types (Fig. 1B). The inflammatory response and cytotoxicity of these CAR-T cells showed target specificity to either CD19 or CD22 (Fig. 1C–E). Subsequently, in the NOG Nalm6 xenograft model, our findings underscored that the bi-specific CD19-CD22 CAR-T (3.0 × 10^5^ cells/mouse) markedly enhances therapeutic outcomes compared to single-target alternatives (CD19 or CD22 CAR-T) or negative controls (Fig. 2). Collectively, these data highlight the potential of bi-specific CD19-CD22 CAR-T as a potent therapeutic avenue for r/r B-ALL patients.

In addition to its pronounced antitumor efficacy, the bi-specific CD19-CD22 CAR-T cell infusion was associated with several severe adverse events. Notably, CRS events were observed in 41.94% of r/r B-ALL patients (13/31, SI Table S3), with only one patient experiencing a Grade-3 CRS (Fig. 3). On subsequent monitoring, this patient progressed to a state of progressive disease (PD) and subsequently withdrew from the trial. Concurrently, no CRES events were reported among these r/r B-ALL patients (SI Table S3). Pertaining to other adverse events, certain manifestations such as blood and lymphatic system disorders, fever, malaise, fatigue, hypokalemia, and hypocalcemia were notably more prevalent, warranting closer scrutiny in subsequent clinical trials (SI Table S4, S5). This safety profile might be attributed to the bi-specific CAR-T cell infusion, emphasizing the need for further clinical investigations using varied CAR-T cell infusions.

To assess the in vivo antitumor efficacy of the bi-specific CD19-CD22 CAR-T, we initiated a phase I trial involving patients diagnosed with r/r B-ALL. Of the 35 patients enlisted, 31 underwent the full protocol involving CAR-T infusion followed by data collection. Remarkably, within a month, 29 of the 35 participants (82.86%) reached complete remission (CR) as depicted in Fig. 4, 5 and SI Fig. S4. Taking stem cell transplantation into account, a discernible difference was noted: the complete remission rate for r/r B-ALL patients who underwent transplantation (13/16) substantially outpaced those who did not (2/15). Furthermore, our analysis of clinical baseline characteristics in correlation with CR indicated that most baseline metrics (Figs. 6, 7 and SI Table 1–6) did not significantly differ between CR and non-CR patients. Exceptions included the abundance of CD19 and CD22, along with IFN-*γ* levels. Notably, metrics such as CD19_Tmax_, CD19_AUC0-28_, CD22_Cmax_, AUC0-28, and IFN-*γ*_Tmax_ were markedly elevated in CR patients compared to their non-CR counterparts. Elevated levels of CD19 and CD22 imply that the preservation of target antigens might be pivotal for achieving optimal clinical outcomes, suggesting these parameters might serve as future biomarkers to guide therapeutic interventions. It is worth noting, however, that previous studies have not consistently linked IFN-*γ* levels with differentiation between CR and non-CR states, highlighting the need for further meticulous clinical scrutiny.

A limitation of our clinical study is the absence of comparator groups, specifically those receiving CD19 CAR-T cell infusion, CD22 CAR-T cell infusion, or a combination of CD19/CD22 CAR-T cell infusions, which would elucidate therapeutic efficacies in r/r B-ALL patients. While antigen loss stands out as a primary factor for relapsed or refractory progression, there is a paucity of clinical reports comparing the bi-specific CD19-CD22 CAR with single-target CAR-T cell therapies [24]. Future studies should examine the relationship between baseline characteristics, inflammatory markers, and other parameters to underscore the significance of bi-specific CD19-CD22 CAR relative to other CAR therapies [24].

## Conclusion

Our study underscores the feasibility, safety, and marked efficacy of bi-specific CD19-CD22 CAR-T in enhancing the survival of r/r B-ALL patients. This dual-targeted approach exhibited significant persistence in vivo, durable responses, and efficient clearance of r/r B-ALL malignancies. Both our Nalm6 xenograft model and preliminary clinical outcomes corroborate this antitumor efficacy. In our clinical trial, involving 35 r/r B-ALL patients, we observed a connection between the bi-specific CAR-T cell treatment and patient baseline characteristics. The full spectrum of benefits offered by the bi-specific CD19-CD22 CAR-T treatment merits a deeper exploration, and its efficacy in conjunction with other variables warrants examination in subsequent clinical studies.

## Methods and materials

### Clinical protocol design

We initiated a clinical trial to assess the therapeutic potential of CD19-CD22 CAR-T in patients with relapsed or refractory B-ALL. The objective of this study was to determine the safety and efficacy of this dual-targeted therapy. Eligibility criteria and other pertinent details regarding patient enrollment can be found at the clinical trial repository (https://clinicaltrials.gov/ct2/show/NCT04303520):

*Inclusion Criteria:* Eligible patients are male or female between 13 and 70 years old, with an ECOG score of 0–2, and histologically confirmed CD19-positive lymphoma, who have received at least two prior combination chemotherapy regimens or experienced disease recurrence. Patients must also meet specific laboratory and health condition requirements, such as creatinine levels < 2.5 mmol/l and cardiac ejection fraction > 50%.

*Exclusion Criteria:* Patients are excluded if they have other malignancies, active infections (such as hepatitis B, C, or HIV), severe cardiovascular or respiratory disease, uncontrolled infections, or have previously received CAR-T therapy. Pregnant or lactating women, and those deemed inappropriate for the trial, are also excluded.

The trial protocol received approval from the Institutional Review Board of Henan Province Hospital of Traditional Chinese Medicine (The Second Affiliated Hospital of Henan University of Chinese Medicine), Zhengzhou, China. All participants in the study were administered a single dose of CD19-CD22, with dosage levels ranging between 1 to 12 × 10^6^ cells/kg (SI Table S8). Our evaluation metrics encompassed dose-limiting toxicity (DLT), adverse event occurrences (AE), overall response rate (ORR), complete remission (CR) rate, overall survival (OS) rate, serum cytokine concentrations, and cellular peak kinetics.

### CD19-CD22 CAR construct and manufacturing of CD19-CD22 CAR-T cells

CD19-CD22 dual-target CAR lentiviral construct was designed by containing CD19 and CD22 single-chain variable fragments. These fragments were driven from clone FMC63 and m971, respectively. Lentivirus used was produced using 293 T cells and then extracted. All lentivirus was stored at − 80 °C.

### Generation of retroviral vectors

The constructed anti-CD19/CD22 CAR encompassed a signal peptide, anti-CD19 and anti-CD22 scFvs, a CD8*α* hinge coupled with a transmembrane domain, the 4-1BB co-stimulatory signaling domain, and the CD3 *ζ* cytoplasmic region. Codon-optimized cDNA sequences encoding this CAR were synthesized by Integrated DNA Technologies (Coralville, IA) and subsequently cloned into a retroviral vector. This vector was derived from a modified Moloney murine leukemia virus using established molecular cloning methodologies [38]. Clinical-grade retroviral producer cell lines, essential for CAR vector production, were established utilizing the PG13 gibbon ape leukemia virus packaging cell line (CRL-10686, ATCC), as detailed in prior work [39].

### Cell culture

Cell lines K562-CD19, K562-CD22, and K562 cell lines, Raji and Nalm6 were obtained from the ATCC (Rockville, MD). Cells were maintained in RPMI-1640 media (#11875-093, Gibco) containing 10% fetal bovine serum (FBS) (35–076-CU, Corning), whereas 293 T cells were maintained in DMEM (10–017-CV, Corning) containing 10% FBS.

### Preparation of anti-CD19/CD22 CAR-T cells

Thawed PBMCs from healthy donors were cultured in T cell medium (TCM) containing X-vivo15 serum-free medium (Lonza, Allendale, NJ), 5% (vol/vol) GemCell human serum antibody AB (Gemini Bio Products, West Sacramento, CA), 1% (vol/vol) Glutamax-100 (GIBCO Life Technologies), 10 mM HEPES buffer (Corning), 1% (vol/vol) penicillin/streptomycin (Corning), and 12.25 mM N-acetyl-L-cysteine (Sigma). The culture was supplemented with 50–100 IU/mL human IL-2. The PBMCs were activated and expanded using Dynabeads human T-expander CD3/CD28 (Invitrogen) at a bead:PBMC ratio of 1:1. The retroviral supernatants were spin-loaded onto non-tissue culture-treated 12-well plates coated with 15-mg retronectin (Clontech Laboratories, Mountain View, CA) per well by centrifuging 2 h at 2000 rpm at 32 °C. Activated PBMCs were resuspended at the concentration of 10^6^ cells/mL in TCM, containing 50 IU/mL recombinant human IL-2, and then added to the vector-loaded 12-well plate. The plates were spun at 600 g at 32 °C for 30 min and incubated at 37 °C overnight. The media was replenished to keep cell densities between 0.5 and 1.5 × 10^6^ cells/mL. During ex vivo expansion, culture medium was replenished, and T cell density was maintained between 0.5 and 1 × 10^6^ cells/mL.

### Flow cytometry measurement

For cell surface staining, the following antibodies were used: APC/Cy7-conjugated mAb against human CD3 (BioLegend), APC-conjugated mAb against mouse Fab (BioLegend), biotin goat anti-mouse F(abʹ)2 fragment, and mAb against CD107a (E Biosciences). T cells were stained with above antibodies for 20 min at 4 °C before flow cytometry assay. For intracellular staining, T cells were fixed and permeabilized with fixation/permeabilization solution (51-2090KZ, BD Biosciences) according to the manufacturer’s protocol. The ability of CAR-T cells to produce IFN-*γ* upon B-ALL cell stimulation was assessed by using PE mouse mAb against human IFN-*γ* (BD Biosciences). All samples were acquired on a flow cytometer (MACSQuant^®^Analyzer 10, Miltenyi Biotec, Germany).

### Cytotoxicity assay

As previously described, a flow cytometry-based T cell cytotoxicity assay was employed to measure the cytotoxic response of CAR-T cells in the presence of target cells [40]. Briefly, anti-CD19/CD22 CAR-T cells were incubated with CFSE-labeled tumor cells at various E: T ratios (5:1, 1:1). After 16 h, cells were harvested, stained for 7-ADD, and subjected to flow cytometry analysis (MACSQuant^®^Analyzer 10).

### Pharmacodynamics studies in vivo

NOG mice (NOD/Shi-scid/IL-2R*γ*^null^) were purchased from Beijing Vital River Laboratory Animal Technology Co., Ltd. Mice were housed under pathogen-free conditions with a 12-h light/dark cycle and had free access to food and water (Shanghai Model Organisms Center, Inc.). For pharmacodynamic study, 6-week-old female NOG mice were injected with 5 × 10^5^ Nalm6 tumors cells via tail vein at − 3 day. Mice were then injected *i.v.* with 3 × 10^6^ CAR + cells in 0.9% NaCl solution, while control mice were injected *i.v.* with NT cells in 0.9% NaCl solution.

### Measurement of cell-free tumor DNA

DNA was extracted from patient blood samples. PCR amplification of the biomarkers mentioned in the context was performed using QIAamp DNA Mini Kit (catalog no. 51306; QIAGEN) at baseline and mentioned days in the figures. CAR presence was measured by RT-qPCR with using primer and probe sequences according to previous reports.

### Cytokine measurement

Target cells mentioned in the context were seeded at 2 × 10^4^ cells per well in 96-well plates, and then incubated with relative effector cells (CD19, CD22, and CD19-CD22 CAR-T cells, respectively) at an E;T ratio of 1:1 for 16 h (*n* = 3 replicates per group). The supernatants were harvested to examine the excreted levels of cytokines (including CD107a and IFN-*γ*).

### Quantitative PCR

The expansion and persistence of CD19-CD22 CAR-T cells in the blood were examined by q-PCR method following our previous reports [41]. Briefly, genomic DNA of patient samples was extracted using MiniBEST Universal Genomic DNA Extraction Kit (Takara) and then stored at −80 °C until for further examination. The primer sequence for CD19 and CD22 were used for final qPCR testing:

1922-CD19 probe FAM: TCCTGGATCAGACAGC.

- FP: TGTCCCTGCCCGATTACG
- RP: AGCCATTCCAGTCCCTTTCTG

1922-CD22 probe FAM: CCTGACAATTAGCAGTCTG.

- FP: CAGCGGCACCGATTTCA
- RP: GGCAATAGTAGGTGGCGAAGTC

### Biomarker examination

Levels of serum cytokines mentioned in the manuscript were tested in the clinic. Briefly, collected blood samples were cryopreserved and delivered back to the central facility for other measurement by our previous report [41].

### Statistical analysis

All the images were generated using GraphPad Prism version 7.0 software. All the data were presented as Means ± SEM. Statistical analysis was conducted using approaches as descripted in figure captions. P value is less than 0.05 considered as significance. 

## Supplementary Information

Below is the link to the electronic supplementary material.

Supplementary file1 (DOCX 1148 KB)

## Data Availability

The original contributions presented in the study are included in the article/supplementary material. Further inquiries can be directed to the corresponding author.
