# Supplementary material for: A bi-specific CAR-T cell therapy targeting CD19 and CD22 in relapsed or refractory B-ALL
Source: Clin Exp Med. 2025 Jul 28;25(1):264. doi: 10.1007/s10238-025-01637-8 (PMC12304051; doi:10.1007/s10238-025-01637-8)
Supplement: Supplementary file 1 — Supplementary file1 (DOCX 1148 KB) [file 10238_2025_1637_MOESM1_ESM.docx]

**A bi-specific CAR-T cell therapy targeting CD19 and CD22 in relapsed or refractory B-ALL**

Qiuling Ma^1,2^, Runhong Wei^2^, Qingming Wang^3^, Songfu Jiang^4^ ,Yi Wu^2^, Min Chao^3^, Fangshu Guo^3^, Yu Zhang^4^, Xiaohong Sun^4^, Haigang Wu^5,*^, Fang Xiang^6^, Lin Zhun^6^, Zhi Cheng^1,2*^

1. The Second School of Clinical Medicine, Henan University of Chinese Medicine, Zhengzhou 450046, P.R.China.

2. Department of Hematology, Henan Province Hospital of Traditional Chinese Medicine (The Second Affiliated Hospital , Henan University of Chinese Medicine), Institute of Hematology, Henan University of Chinese Medicine, Zhengzhou 450002, P.R.China.

3. Department of Hematology, The Second Affiliated Hospital of Nanchang University, Nanchang, Jiangxi,330006, P.R.China.

4. Department of Hematology, The First Affiliated Hospital of Wenzhou Medical University, Wenzhou, Zhejiang 325000, P.R.China.

5. School of Life Sciences, Henan University, Kaifeng City, 475000, P.R.China

6. Hrain Biotechnology Co. Ltd., Shanghai 200030, P.R.China

## Supplementary information


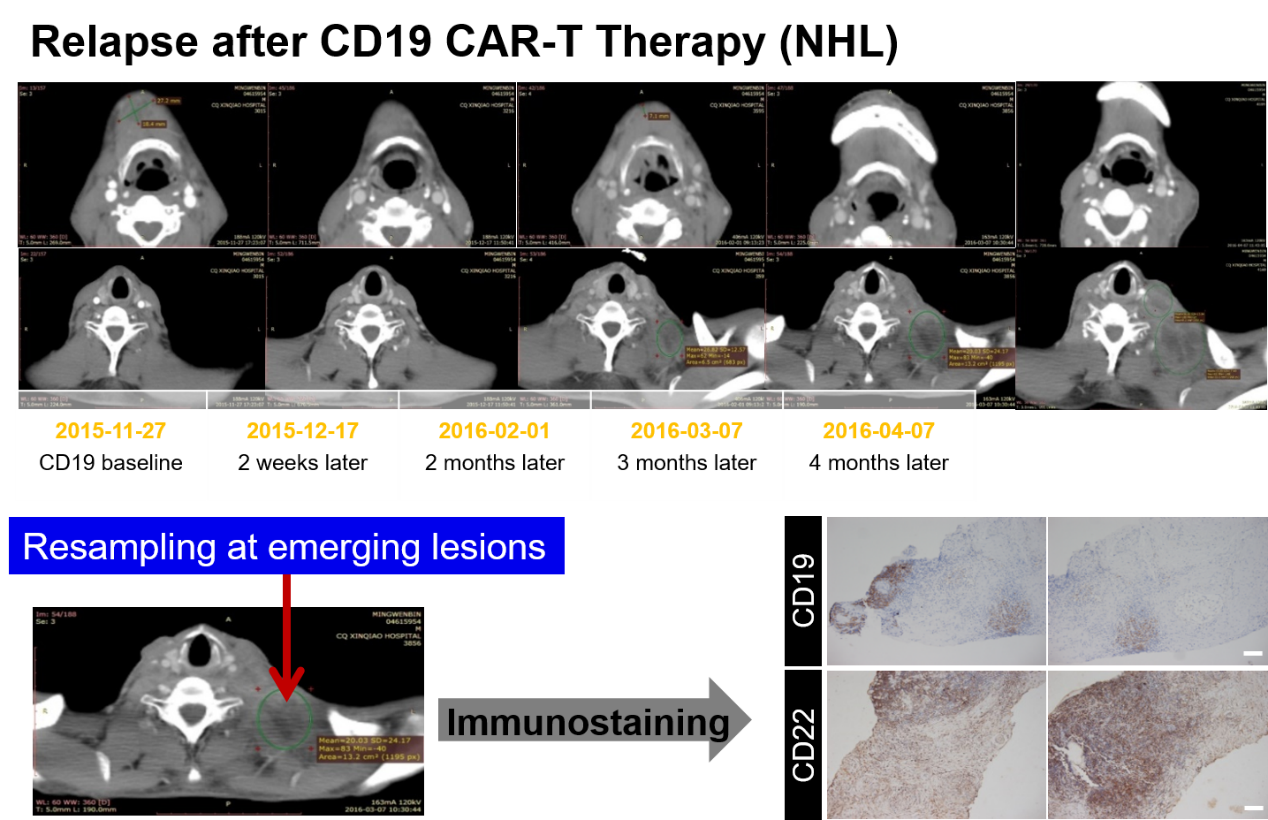


SI Fig.S1. CD22 expression levels is significantly increased post CD19 CAR-T therapy at 4 months. Expression levels of CD22 were stained by IHC method. Scale bar is 200 μm.

SI Table S1. Proliferation of CAR-T incubated with targeting cell lines.

| Group | T Cell donor | Mixture | Proliferation |
| --- | --- | --- | --- |
| 1 | Donor-1 | NT | 0.21 |
| 2 |  | NT+Raji | 0.35 |
| 3 |  | CD19-CD22 CAR-T | 0.19 |
| 4 |  | CD19-CD22 CAR-T+Raji | 1.39 |
| 5 | Donor-2 | NT | 0.018 |
| 6 |  | NT+Raji | 0.010 |
| 7 |  | CD19-CD22 CAR-T | 0.060 |
| 8 |  | CD19-CD22 CAR-T+Raji | 1.201 |
| 9 | Donor-3 | NT | 0.028 |
| 10 |  | NT+Raji | 0.12 |
| 11 |  | CD19-CD22 CAR-T | 0.39 |
| 12 |  | CD19-CD22 CAR-T+Raji | 2.56 |

NT: nature T cells, originated from donors.


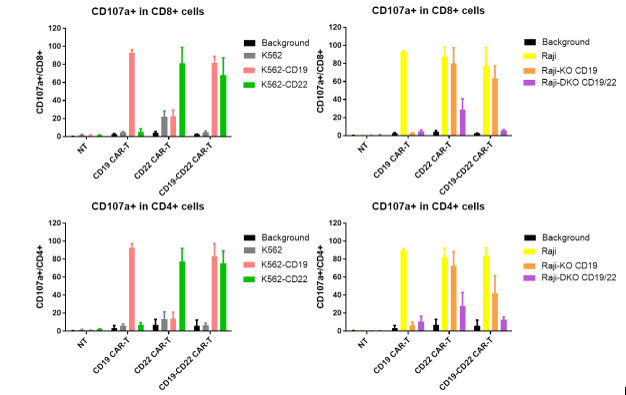


SI Figure S2. Production of CD107a by CD8+ and CD4+ T cells from CD19-CD22 CAR-T group (mixture ratio 1:1) after incubation with K562, K562-CD19, K562-CD22 (left panel), and Raji, Raji-KO CD19, Raji-DKO CD19/CD22 cells (right panels). Data were presented as Mean ± Standard Error. N=3 replicates per group.


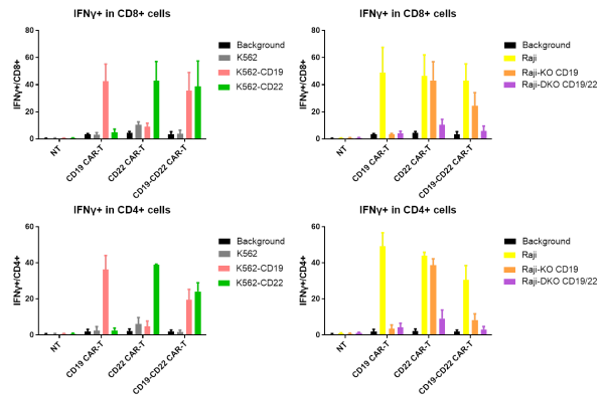


SI Figure S3. Production of IFN-γ by CD8+ and CD4+ T cells from CD19-CD22 CAR-T group (mixture ratio 1:1) after incubation with K562, K562-CD19, K562-CD22 (left panel), and Raji, Raji-KO CD19, Raji-DKO CD19/CD22 cells (right panels). Data were presented as Mean ± Standard Error. N=3 replicates per group.

Table S1. Clinical Characteristics of Patients Following CAR-T Therapy.

| Baseline characteristics | | Ps (N=35) | ≤21 (N=16) | >21 (N=19) |
| --- | --- | --- | --- | --- |
| Median age, year | | 26 (4-60) | 14 (4-21) | 43 (23-60) |
| Male | | 11 (31.43%) | 6 (17.14%) | 5 (14.29%) |
| Female | | 24 (68.57%) | 10 (28.57%) | 14 (40.00%) |
| ECOG score | |  |  |  |
|  | 0-1 | 22 (62.86%) | 9 (25.71%) | 13 (37.15%) |
|  | 2 | 5 (14.29%) | 3 (8.57%) | 2 (5.72%) |
|  | 3 | 3 (8.57%) | 1 (2.86%) | 2 (5.71%) |
|  | 4 | 3 (8.57%) | 2 (5.72%) | 1 (2.86%) |
| Philadelphia chromosome (+) | | 7 (20%) | 1 (2.86%) | 6 (17.14%) |
| Gene mutation (+) | | 12 (34.29%) | 8 (22.86%) | 4 (11.43%) |
| n.t. | | 12 (34.29%) | 6 (17.14%) | 6 (17.14%) |
| Prior stem cell transplants | | 1 (2.86%) | 0 | 1 (2.86%) |
| Percentage Juvenile cell >20% | | 22 (62.86%) | 14 (40%) | 8 (22.86%) |
| CD19^+^CD22^-^ | | 8 (22.86%) | 4 (11.43%) | 4 (11.43%) |
| CD19^-^CD22^+^ | | 1 (2.86%) | 0 | 1 (2.86%) |
| CD19^+^CD22^+^ | | 20 (57.14%) | 10 (28.57%) | 10 (28.57%) |

Notes: Ps, patients; ECOG, the Eastern Cooperative Oncology Group; n.t., no testing.

Table S2. Relationship between baseline characteristics and response.

|  | Ps with CR or Cri.n | Ps with SD, NR or PD.n | p value |
| --- | --- | --- | --- |
| **Overall** | 31 | 4 |  |
| **Sex** |  |  |  |
| Female | 20 | 4 |  |
| Male | 11 | 0 | 0.285 |
| **Age (Years)** |  |  |  |
| ≤21 | 15 | 1 |  |
| ≥21 | 16 | 3 | 0.37 |
| **CD19 (%)** |  |  |  |
| >1 | 25 | 4 |  |
| ≤1 | 1 | 0 | 0.627 |
| **CD22 (%)** |  |  |  |
| >1 | 17 | 4 |  |
| ≤1 | 8 | 0 | 0.222 |
| **Blasts in bone marrow at baseline**  **(%)** |  |  |  |
| >20 | 20 | 2 |  |
| ≤20 | 9 | 1 | 0.458 |
| **Philadelphia chromosome** |  |  |  |
| Yes | 5 | 2 |  |
| No | 16 | 0 | 0.11 |
| **ECOG** |  |  |  |
| 0-1 | 22 | 2 |  |
| 2 | 4 | 1 |  |
| 3 | 2 | 1 |  |
| 4 | 3 | 0 | 0.494 |

Notes: Ps, patients; ECOG, the Eastern Cooperative Oncology Group; Cri.n, complete remission (CR)/CR with incomplete blood count recovery; PD.n, progressive disease.

SI Table S3. Adverse Events (n=35)

| Condition | Event types | Total | Grade 1 | Grade 2 | Grade 3 | Grade 4 |
| --- | --- | --- | --- | --- | --- | --- |
|  | CRS | 13 (41.94%) | 6 (19.35%) | 6 (19.35%) | 1  (3.23%) |  |
|  | CRES | 0(0%) | 0(0%) | 0(0%) | 0(0%) | 0(0%) |
| Blood and lymphatic system disorders | Neutropenia | 31 (96.88%) |  | 2  (6.25%) | 5  (15.63%) | 24  (75%) |
|  | Leucopenia | 31  (96.88%) |  | 3  (9.38%) | 3  (9.38%) | 25  (78.13%) |
|  | Lymphocytopenia | 29 (90.63%) | 1  (3.13%) | 1  (3.13%) | 4 (12.5%) | 23  (71.88%) |
|  | Thrombocytopenia | 26  (81.25%) | 1  (3.13%) |  | 2  (6.25%) | 23  (71.88%) |
|  | Febrile neutropenia | 2  (6.25%) |  |  |  | 2  (6.25%) |
|  | Leukocytosis | 1  (3.13%) | 1  (3.13%) |  |  |  |
|  | Lymphocythemia | 1  (3.13%) |  | 1  (3.13%) |  |  |
|  | Anemia | 30 (93.75%) | 3  (9.38%) | 1  (3.13%) | 21 (65.66%) | 5 (15.63%) |
| Infections and infestations | Infection | 1  (3.13%) |  | 1  (3.13%) |  |  |
|  | Pulmonary infection | 2  (6.25%) |  |  | 2  (6.25%) |  |
|  | Pneumonitis | 2(6.25%) | 1 (3.13%) | 1 (3.13%) |  |  |
|  | Upper respiratory infection | 10 (31.25%) | 6 (18.75%) | 3  (9.38%) | 1  (3.13%) |  |
| General disorders and administrations site conditions | Fever | 25 (78.13%) | 5 (15.63%) | 13 (40.63%) | 6 (18.75%) | 1 (3.13%) |
|  | Edema limbs | 1(313%) | 1(3.13%) |  |  |  |
|  | Malaise | 19 (59.38%) | 13  (40.63%) | 6  (18.75%) |  |  |
|  | Rigor | 1  (3.13%) |  | 1 (3.13%) |  |  |
|  | Fatigue | 17 (53.13%) | 13 (40.63%) | 4 (12.5%) |  |  |
|  | Pain | 6  (18.75%) | 5  (15.63%) | 1 (3.13%) |  |  |
| Respiratory,thoracic  And mediastinal disorders | Oropharyngeal pain | 3 (9.38%) | 3(9.38%) |  |  |  |
|  | Pleural effussion | 1(3.13%) | 1(3.13%) |  |  |  |
|  | Chest pain | 1(3.13%) |  | 1(3.13%) |  |  |
|  | Hypoxia | 1(3.13%) |  |  | 1(3.13%) |  |
|  | Hemorrhinia | 2  (6.25%) |  | 2 (6.25%) |  |  |
|  | Cough | 6  (18.75%) | 4 (12.5%) | 2 (6.25%) |  |  |

Notes: “Absolute Neutrophil Count, NEUT; Total Lymphocyte Count, TLC; Platelet Count, PC; Febrile Neutropenia, FN; White Blood Cell, WBC.

Table S4. Adverse Events (continue)

| Condition | Event types | Total | Grade 1 | Grade 2 | Grade 3 | Grade 4 |
| --- | --- | --- | --- | --- | --- | --- |
| Gastrointestinal disorders | Recurrent aphthous ulcer | 1 (3.13%) | 1  (3.13%) |  |  |  |
|  | Dry mouth | 2 (6.25%) | 2  (6.25%) |  |  |  |
|  | Abdominal distention | 11 (34.38%) | 9  (28.13%) | 2 (6.25%) |  |  |
|  | Nausea | 15 (46.88%) | 12  (37.5%) | 3 (9.38%) |  |  |
|  | Abdominal pain | 1 (3.13%) | 1  (3.13%) |  |  |  |
|  | Constipation | 1 (3.13%) | 1  (3.13%) |  |  |  |
|  | Anal pain | 1 (3.13%) | 1  (3.13%) |  |  |  |
|  | Diarrhea | 1 (3.13%) |  | 1 (3.13%) |  |  |
|  | Vomiting | 2 (6.25%) | 1  (3.13%) | 1 (3.13%) |  |  |
|  | Dysphagia | 6 (18.75%) | 1  (3.13%) | 3 (9.38%) | 2 (6.25%) |  |
| Eye disorders | Blurred vision | 1 (3.13%) | 1  (3.13%) |  |  |  |
|  | Periorbital edema | 1 (3.13%) | 1  (3.13%) |  |  |  |
| Immune system disorders | Allergy | 4 (12.5%) | 3  (9.38%) | 1(3.13%) |  |  |
| Vascular disorders | Hypertension | 6 (18.75%) | 5  (15.63%) |  | 1 (3.13%) |  |
|  | Hypotension | 13 (40.63%) | 11 (34.38%) | 1 (3.13%) | 1 (3.13%) |  |
| Psychiatric disorders | Hallucination | 1(3.13%) | 1 (3.13%) |  |  |  |
| Nervous system disorders | Headache | 2  (6.25%) | 1  (3.13%) | 1  (3.13%) |  |  |
|  | Hypersomnia | 1  (3.13%) |  | 1 (3.13%) |  |  |
| Cardiac disorders | Palpitation | 1 (3.13%) | 1  (3.13%) |  |  |  |
|  | Pericardial effusion | 2 (6.25%) |  | 2 (6.25%) |  |  |
| Hepatobiliary disorders | Liverdamage | 1 (3.13%) |  | 1 (3.13%) |  |  |
| Musculoskeletal  and connective tissue disorders | Arthralgia | 1 (3.13%) | 1  (3.13%) |  |  |  |
|  | Muscle weakness lower limb | 1 (3.13%) | 1  (3.13%) |  |  |  |
|  | Bone pain | 1 (3.13%) |  | 1 (3.13%) |  |  |
| Renal and urinary  disorders | Glucosuria | 1 (3.13%) | 1  (3.13%) |  |  |  |
|  | Proteinuria | 1 (3.13%) |  | 1 (3.13%) |  |  |

Table S5. Adverse Events (continue)

| Condition | Event types | Total | Grade 1 | Grade 2 | Grade 3 | Grade 4 |
| --- | --- | --- | --- | --- | --- | --- |
| Metabolism and nutrition disorders | Hypokalemia | 17 (53.13%) | 9 (28.13%) | 5 (15.63%) | 2 (6.25%) | 1 (3.13%) |
|  | Hypernatremia | 1 (3.13%) | 1  (3.13%) |  |  |  |
|  | Hyponatremia | 7 (21.88%) | 5 (15.63%) | 1 (3.13%) |  | 1  (3.13%) |
|  | Hypocalcemia | 19 (59.38%) | 10 (31.25%) | 6 (18.75%) | 1 (3.13%) | 2  (6.25%) |
|  | Hypomagnesemia | 5 (15.63%) | 5 (15.63%) |  |  |  |
|  | Hypochloraemia | 1 (3.13%) | 1  (3.13%) |  |  |  |
|  | Hypermagnesemia | 1 (3.13%) | 1  (3.13%) |  |  |  |
|  | Hypophosphatemia | 9 (28.13%) | 8  (25%) | 1 (3.13%) |  |  |
|  | Hyperphosphatemia | 9 (28.13%) | 9 (28.13%) |  |  |  |
|  | Hyperuricemia | 3 (9.38%) | 2  (6.25%) |  |  | 1  (3.13%) |
|  | Hypercalcemia | 1 (3.13%) | 1  (3.13%) |  |  |  |
|  | Hyperchloremia | 2 (6.25%) | 2  (6.25%) |  |  |  |
|  | Hyperglycemia | 9 (28.13%) | 9 (28.13%) |  |  |  |
|  | Hypoalbuminemia | 23 (71.88%) | 15 (46.88%) | 6 (18.75%) | 1 (3.13%) | 1  (3.13%) |
|  | Hypertriglyceridemia | 2 (6.25%) |  | 2 (6.25%) |  |  |
| Investigations | Weight loss | 1 (3.13%) |  | 1 (3.13%) |  |  |
|  | Creatinine increased | 2 (6.25%) | 2  (6.25%) |  |  |  |
|  | Blood lactate dehydrogenase increased | 13 (40.63%) | 8  (25%) | 2 (6.25%) | 1 (3.13%) | 2  (6.25%) |
|  | Fibrinogen decreased | 7 (21.88%) | 2  (6.25%) | 3 (9.38%) | 1 (3.13%) | 1  (3.13%) |
|  | GGT increased | 9 (28.13%) | 4  (12.5%) |  | 2 (6.25%) | 3 (9.38%) |
|  | Alkaline phosphatase increased | 8  (25%) | 4  (12.5%) | 1 (3.13%) |  | 3  (9.38%) |
|  | Aspartate aminotransferase increased | 9 (28.13%) | 4  (12.5%) | 1 (3.13%) | 1 (3.13%) | 3  (9.38%) |
|  | Alanine aminotransferase decreased | 1 (3.13%) | 1  (3.13%) |  |  |  |
|  | Aspartate aminotransferase decreased | 1 (3.13%) | 1  (3.13%) |  |  |  |
|  | Alanine aminotransferase increased | 2 (6.25%) | 2  (6.25%) |  |  |  |
|  | Urine output decreased | 1 (3.13%) |  |  | 1 (3.13%) |  |
|  | Activated partial thromboplastin time prolonged | 3 (9.38%) | 3  (9.38%) |  |  |  |
|  | Blood bilirubin increased | 2 (6.25%) | 1  (3.13%) | 1 (3.13%) |  |  |
|  | INR increased | 1 (3.13%) | 1  (3.13%) |  |  |  |

Note: INR, international normalized ratio; Gamma-glutamyl Transferase, GGT.

Table S6. Relationship between baseline characteristics and CR.

| Index | Total | CR patients | No CR patients | *p* value |
| --- | --- | --- | --- | --- |
| CD19 C_max_ | 97249±58913 | 106740±53714 | 37930±62210 | 0.13 |
| CD19 T_max_ | 10 (4-21) | 10 (6-21) | 8 (4-10) | 0.06 |
| CD19 AUC0-28 | 1044025(8790-4464039) | 1088866(361851-4464039) | 36849(8790-1634560) | 0.037 |
| CD22 C_max_ | 93275±53028 | 103389±49473 | 30065±49473 | 0.03 |
| CD22 T_max_ | 11±4 | 11±4 | 8±3 | 0.21 |
| CD22 AUC0-28 | 911182(5154 -3843854) | 1064029(377526-3843854) | 29763(5154-1351199) | 0.023 |
| IL6 T_max_ | 5(0-24) | 5(0-24) | 3.5(1-10) | 0.44 |
| IL6_Peak_ | 140(23-66530) | 137(35-17184) | 203(23-66530) | 0.78 |
| CRP T_max_ | 7(1-28) | 7(1-28) | 7(3-10) | 0.927 |
| CRP_Peak_ | 203.2(13-16478) | 203(13-16478) | 324(42-641) | 0.604 |
| IL15 T_max_ | 1(0-9) | 1(0-9 | 2(1-4) | 0.831 |
| IL15_Peak_ | 18.62(5.45-24955) | 16(9-24955) | 29(5-125) | 0.562 |
| TNF-α T_max_ | 5(1-28) | 6(1-28) | 3.5(3-28) | 0.927 |
| TNF-α_Peak_ | 5.9(0.4-28.7) | 6(0.4-29) | 4(1.6-6.5) | 0.142 |
| IFN-γ T_max_ | 6(1-28) | 7(1-28) | 3.5(1-7) | 0.043 |
| IFN-γ_Peak_ | 31.5(3.92-4484) | 28(4-4484) | 53(13-575) | 0.341 |
| GranzymeB T_max_ | 7(4-31) | 7(5-31) | 7(4-10) | 0.692 |
| GranzymeB _Peak_ | 470.1(35.7-33947) | 620(36-33947) | 158(36-5951) | 0.482 |

Detailed information of patients with NR or PD

**Patient 1**: Female, 60 year-old, body weight 71 kg, dosage of CAR-T infusion is 6×10^6^ cells/kg; BCR-ABL1(p210) positive; abundance of Juvenile cells is 60.50%; percentage of CD19^+^ and CD22^+^ in PB cells is 62.20% and 62.70%, respectively; ECOG score is 1; CRS is Grade 3; patient received Tocilizumab therapy and hormone therapy. Before CAR T-cell infusion, patient received chemotherapy, including VDP+TKI, VDP+TKI, CAM, and HperCVAD.

**Patient 2**: Female, 57 year-old, body weight 50 kg, dosage of CAR-T infusion is 6×10^6^ cells/kg; BCR-ABL1(p210) positive; abundance of Juvenile cells is 19.50%; percentage of CD19^+^ and CD22^+^ in PB cells is 2.38% and 1.12%, respectively; ECOG score is 3; CRS is Grade 2. Before CAR T-cell infusion, patient received chemotherapy, including VDP+TKI, VDP+TKI, CAM, and HperCVAD.

**Patient 3**: Female, 16 year-old, body weight 50 kg, dosage of CAR-T infusion is 6×10^6^ cells/kg; abundance of Juvenile cells is 97.00%; percentage of CD19^+^ and CD22^+^ in PB cells is 47.30% and 2.90%, respectively; ECOG score is 2. Before CAR T-cell infusion, patient received sequential chemotherapy as follows:

1) VDCP;

2) Hyper-CVAD A;

3) Hyper-CVAD B+ Pegaspargase;

4) CAM;

5) Hyper-CVAD A;

6) Hyper-CVAD B;

7) VDCLP;

8) Hyper-CVAD B+ Pegaspargase;

9) VP.

**Patient 4**: Female, 51 year-old, body weight 51 kg, dosage of CAR-T infusion is 6×10^6^ cells/kg; percentage of CD19^+^ and CD22^+^ in PB cells is 66.70% and 63.90%, respectively; ECOG score is 1. Before CAR T-cell infusion, patient received sequential chemotherapy as follows:

1) CHOP*6;

2) ECHOP;

3) CAR-T therapy；

4) FCD;

5) Chlorambucil;

6) FC*3.


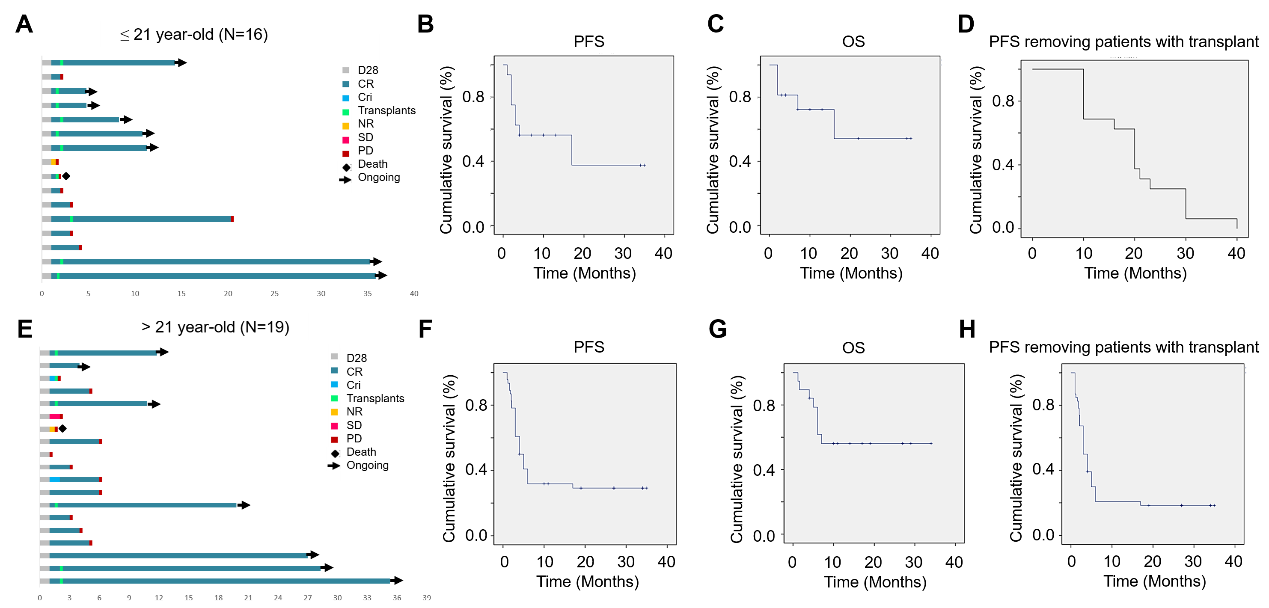


SI Fig.S4. **Clinical outcome of bi-specific CD19-CD22 CAR T-cell infusion depended on age (threshold value is 21 year-old)**. **A**, Swimmer plot for the 16 patients receiving the bi-specific CD19-CD22 CAR T-cell infusion in this investigation (age is less than 21). **B**, Progression-free survival post bi-specific CD19-CD22 CAR T-cell infusion for B-ALL patients (age is less than 21). Median survival is 17.00 ± 12.06 months, 95% CI (0.00-40.64). **C**, Overall survival post bi-specific CD19-CD22 CAR T-cell infusion for B-ALL patients (age is less than 21). Average survival is 22.85 ± 4.36 months, 95% CI (14.31-31.40). **D**, Progression-free survival post bi-specific CD19-CD22 CAR T-cell infusion for B-ALL patients (age is less than 21) without stem cell transplantation. Median survival is 2.0 ± 0.19 months, 95% CI (1.62-2.38). **E**, Swimmer plot for the 19 patients receiving the bi-specific CD19-CD22 CAR T-cell infusion in this investigation (age is more than 21). **F**, Progression-free survival post bi-specific CD19-CD22 CAR T-cell infusion for B-ALL patients (age is more than 21). Median survival is 4.00 ± 0.74 months, 95% CI (2.55-5.48). **G**, Overall survival post bi-specific CD19-CD22 CAR T-cell infusion for B-ALL patients (age is more than 21). Average survival is 21.13 ± 3.43 months, 95% CI (14.41-27.85). **H**, Progression-free survival post bi-specific CD19-CD22 CAR T-cell infusion for B-ALL patients (age is more than 21) without stem cell transplantation. Median survival is 3.0 ± 0.50 months, 95% CI (2.03-3.97). Data were estimated using Kaplan-Meier method for 16- or 19-patient cohort.

SI Table S7. Clinical outcome analysis of CART-cell infusion depended on age.

| Age | Value | ORR rate | ORR rate  In 3 month | Better ORR rate  In 3 month |
| --- | --- | --- | --- | --- |
| >21 year-old |  | 84.21% | 61.5% | 76.9% |
|  | 95% CI | 68.3%-99.7% | 36.5%-87.5% | 53.5%-100.5% |
|  | CRS rate | 36.84% | 46.15% | 46.15% |
| ≤21 year-old |  | 93.75% | 28.6% | 85.7% |
|  | 95% CI | 82%-105.5% | -4.3%-62.3% | 60.5%-111.5% |
|  | CRS rate | 50% | 57.14% | 57.14% |

Table S8. Administration dose of CAR-T for patient in this study;

| Patient | Dose  (x10^6^ cell) | Patient | Dose  (x10^6^ cell) | Patient | Dose  (x10^6^ cell) |
| --- | --- | --- | --- | --- | --- |
| 1 | 6 | 14 | 6 | 27 | 3 |
| 2 | 6 | 15 | 6 | 28 | 3 |
| 3 | 6 | 16 | 6 | 29 | 2 |
| 4 | 6 | 17 | 6 | 30 | 2 |
| 5 | 6 | 18 | 6 | 31 | 2 |
| 6 | 6 | 19 | 6 | 32 | 2 |
| 7 | 6 | 20 | 12 | 33 | 1 |
| 8 | 6 | 21 | 12 | 34 | 1 |
| 9 | 6 | 22 | 8 | 35 | 1 |
| 10 | 6 | 23 | 8 |  |  |
| 11 | 6 | 24 | 6 |  |  |
| 12 | 6 | 25 | 4 |  |  |
| 13 | 6 | 26 | 3 |  |  |
